# Supplementary material for: Comprehensive Annotation and Functional Exploration of MicroRNAs in Lettuce
Source: Front Plant Sci. 2021 Dec 24;12:781836. doi: 10.3389/fpls.2021.781836 (PMC8739914; doi:10.3389/fpls.2021.781836)
Supplement: Supplementary file 2 [file Data_Sheet_8.ZIP › GSM2230747.plot/Lsa-miRN1655_Lsat_1_v5_gn_1_86381.1_430_TPlot.pdf]

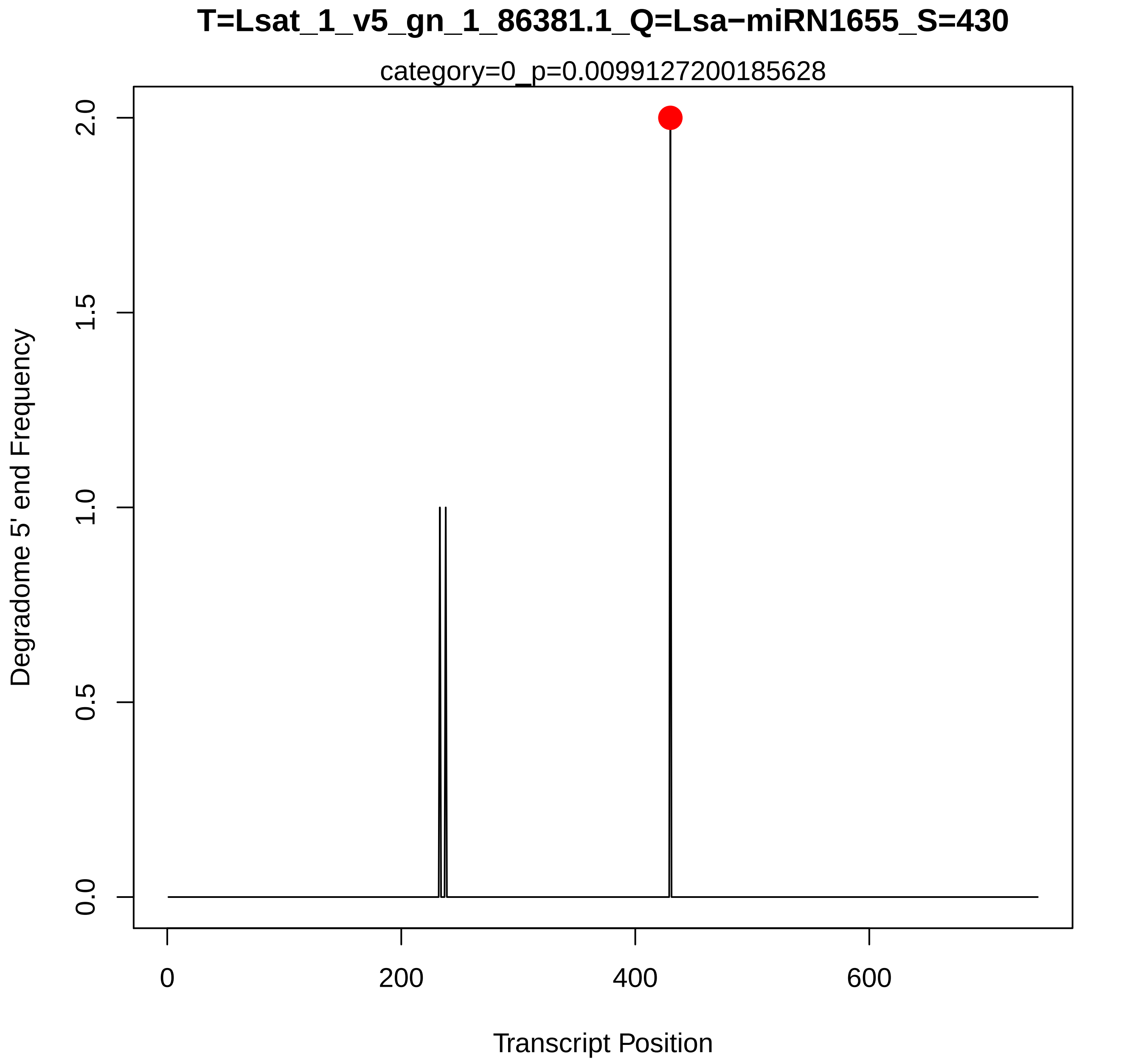

T=Lsat_1_v5_gn_1_86381.1_Q=Lsa−miRN1655_S=430
category=0_p=0.0099127200185628
Degradome 5' end Frequency
0.0
0.5
1.0
1.5
2.0
0
200
400
600
Transcript Position
